# A case of acute onset postoperative gas gangrene caused by *Clostridium perfringens*

**DOI:** 10.1186/s13104-016-2194-0

**Published:** 2016-08-03

**Authors:** Tomonori Takazawa, Jou Ohta, Tatsuo Horiuchi, Hiroshi Hinohara, Fumio Kunimoto, Shigeru Saito

**Affiliations:** 1 Department of Anesthesiology, Gunma University Graduate School of Medicine, 3-39-22 Showa-machi, Maebashi, Gunma 371-8511 Japan; 2 Department of Intensive Care, Gunma University Graduate School of Medicine, 3-39-22 Showa-machi, Maebashi, Gunma 371-8511 Japan

**Keywords:** Gas gangrene, *Clostridium perfringens*, Postoperative

## Abstract

**Background:**

Gas gangrene is a necrotic infection of soft tissue associated with high mortality rates. We report a case of postoperative gas gangrene with very acute onset and rapid progression of symptoms. To our knowledge, this case is the most acute onset of postoperative gas gangrene ever reported.

**Case presentation:**

A 65-year-old Japanese female patient developed a shock state 16 h after radical cystectomy with ileal conduit reconstruction. Two days after the operation, she was transferred to the intensive care unit because of deterioration in her respiratory and circulatory condition. Soon after moving her to the ICU, a subcutaneous hemorrhage-like skin rash appeared and extended rapidly over her left side. Blood tests performed on admission to the ICU indicated severe metabolic acidosis, liver and renal dysfunction, and signs of disseminated intravascular coagulation. Suspecting necrotizing fasciitis or gas gangrene, we performed emergency fasciotomy. Subsequently, multidisciplinary treatment, including empirical therapy using multiple antibiotics, mechanical ventilation, hyperbaric oxygen therapy, polymyxin B-immobilized fiber column direct hemoperfusion, and continuous hemodiafiltration, was commenced. Culture of the debris from a wound abscess removed by emergency fasciotomy detected the presence of *Clostridium perfringens*. We hypothesized that the source of infection in this case may have been the ileum used for bladder reconstruction. Although the initial treatment prevented further clinical deterioration, she developed secondary infection from the 3rd week onward, due to infection with multiple pathogenic bacteria. Despite prompt diagnosis and intensive therapy, the patient died 38 days after the operation.

**Conclusion:**

Although the patient did not have any specific risk factors for postsurgical infection, she developed a shock state only 16 h after surgery due to gas gangrene. Our experience highlights the fact that physicians should be aware that any patient could possibly develop gas gangrene postoperatively.

## Background

Gas gangrene is a rare and deadly infection that progresses very rapidly. Although it is usually caused by traumatic injury, it can also occur after surgery [1–5]. While the timing of symptom onset varies among cases, it occurred at least 24 h after surgery in previous reports. Here, we report a case of gas gangrene in which the clinical symptoms of sepsis appeared 16 h after urologic surgery. Although primary care was effective in this case, the patient died 38 days after the surgery. To our knowledge, this case represents the most acute onset of postoperative gas gangrene ever reported.

## Case presentation

**Fig. 1:**
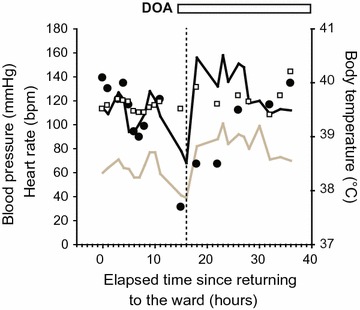
Vital signs of the patient on the day of and after the surgery. The *black* and *brown* lines indicate systolic and diastolic blood pressure, respectively. *Closed circles* and *open squares* indicate body temperature and heart rate, respectively. *DOA* dopamine hydrochloride

**Fig. 2:**
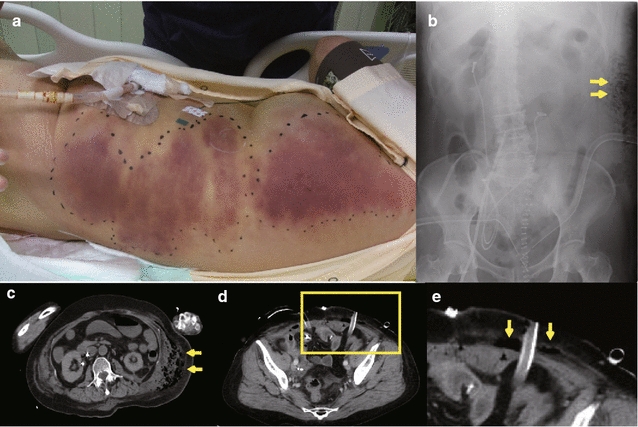
Images of the patient. **a** A diffuse skin rash developed over the* left side* of her trunk 2 days after the surgery. **b** Abdominal X-ray taken immediately after ICU admission showed soft tissue swelling with the density of air on the left abdominal wall (*arrows*). **c** Computed tomography of the abdomen taken 2 days after the surgery showed subcutaneous emphysema over the* left side* of the trunk (*arrows*). **d** Enhanced computed tomography of the abdomen taken 1 day after the surgery. The *rectangle* indicates the area shown in **e**. **e** The *arrows* indicate air-densities between the subcutaneous fat tissue and muscle layer in the vicinity of the left drainage tube

**Fig. 3:**
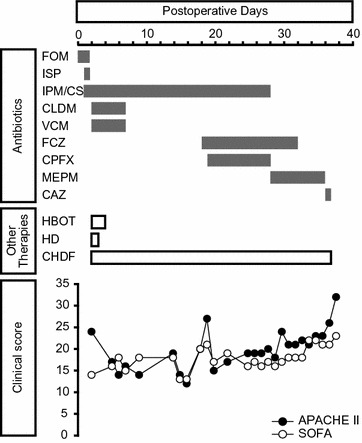
The clinical course of the patient. *FOM* fosfomycin, *ISP* isepamicin, *IPM/CS* imipenem/cilastatin, *CLDM* clindamycin, *VCM* vancomycin, *FCZ* fluconazole, *CPFX* ciprofloxacin, *MEPM* meropenem, *CAZ* ceftazidime, *HBOT* hyperbaric oxygen therapy, *HD* hemodialysis, *CHDF* continuous hemodiafiltration, *APACHE* acute physiology and chronic health evaluation, *SOFA* sequential organ failure assessment

A 65-year-old woman was diagnosed with bladder cancer 6 years earlier, for which she had undergone transurethral resection six times. Oophorectomy for a right ovarian cyst and total hysterectomy for endometriosis had been performed under general anesthesia at the ages of 30 and 35 years, respectively. During the current surgery, she underwent radical cystectomy with creation of an ileal conduit and removal of pelvic lymph nodes. Her past history of multiple laparotomies resulted in intestinal adhesions and massive intraoperative bleeding. The total blood loss during surgery was 5340 ml. She received 1200 ml of autologous blood transfusion, and subsequent transfusion of 560 ml of red cell concentrates and 1200 ml of fresh-frozen plasma in the operation room. The surgical time was 6 h and 49 min. Administration of the antibiotic isepamicin (ISP: 200 mg) was started to treat a fever of 40 °C immediately after moving her to the ward. However, she developed a shock state 16 h after the operation (Fig. 1). Her systolic blood pressure decreased to approximately 70 mmHg and urine output was less than 25 ml/h. Infusions of Ringer’s solution, albumin preparations, immunoglobulins, and vasopressors were started because we suspected septic shock. Administration of imipenem/cilastatin sodium (IPM/CS: 500 mg) was added to ISP because we thought that more intensive empiric antimicrobial therapy was necessary. Two days after the operation, she was transferred to the intensive care unit (ICU) because of deterioration in her respiratory and circulatory condition. Soon after moving her to the ICU, a subcutaneous hemorrhage-like skin rash appeared and extended rapidly over her left side (Fig. 2a). On admission to the ICU, blood tests indicated severe metabolic acidosis, liver and renal dysfunction, and signs of disseminated intravascular coagulation (DIC) (Table 1). Her APACHE II (Acute physiology and chronic health evaluation) and SOFA (Sequential organ failure assessment) scores at this time were 24 and 14, respectively. An X-ray examination and computed tomography (CT), which was performed on postoperative day 2, indicated uninterrupted massive emphysematous tissue from her left chest to lower abdomen (Fig. 2b, c). Suspecting necrotizing fasciitis or gas gangrene, we performed emergency fasciotomy. Subsequently, multidisciplinary treatment, including mechanical ventilation, hyperbaric oxygen therapy (HBOT), polymyxin B-immobilized fiber column direct hemoperfusion (PMX-DHP), and continuous hemodiafiltration (CHDF), was started. Antibiotic therapy was changed to clindamycin (CLDM: 900 mg), vancomycin (VCM: 1000 mg), and IPM/CS (Fig. 3). Gram-positive bacilli, but not Gram-negative bacteria, were detected by microscopic examination of blister fluid aspirated from the skin rash. Moreover, a culture test detected *Clostridium perfringens* (*C. perfringens*) in a wound abscess that was removed during the emergency fasciotomy. Based on these observations, she was diagnosed with gas gangrene. HBOT was performed on the first and second ICU days. We had to abandon plans for a second fasciotomy because the area that required treatment was too large. CHDF was continued through her ICU stay, although PMX-DMP was performed only once on the first ICU day. Her APACHE II and SOFA scores continued to be flat during the first 2 weeks, indicating that the initial treatment prevented further clinical deterioration (Fig. 3). However, she suffered from secondary infection from the 3rd week onward due to infection with multiple pathogenic bacteria, including *Candida albicans* and *Pseudomonas aeruginosa*, as shown in Table 2. Finally, she died of sepsis 38 days after the operation in spite of prompt diagnosis and intensive therapy for the gas gangrene.

**Table 1:** Results of blood tests performed immediately after ICU admission

Blood count	
Hct (%)	28.5
Hb (g/dl)	9.2
WBC (/μl)	7500
Plt (/μl)	7.1 × 10^4^
Blood coagulation tests	
PT (%)	37
APTT (s)	59.3
Fibrinogen (mg/dl)	385
FDP (μg/ml)	47.3
d-dimer (μg/ml)	39.1
TAT (ng/ml)	36.1
PIC (μg/ml)	1.3
Biochemical tests	
T-bil (mg/dl)	6.2
AST (IU/l)	376
ALT (IU/l)	41
LDH (IU/l)	3067
BUN (mg/dl)	49
Cr (mg/dl)	2.23
CRP (mg/dl)	24.24
Blood gas analysis	
pH	7.265
pCO_2_	14.3
pO_2_ (FM 8L)	91.7
HCO_3_^−^ (mmol/l)	10.5
BE (mmol/l)	−20.6

*Hct* hematocrit, *Hb* hemoglobin, *WBC* white blood cells, *Plt* platelets, *PT* prothrombin time, *APTT* activated partial thromboplastin time, *FDP* fibrin/fibrinogen degradation products, *TAT* thrombin-antithrombin complex, *PIC* plasmin-α2 plasmin inhibitor complex, *T-bil* total bilirubin, *AST* aspartate aminotransferase, *ALT* alanine aminotransferase, *LDH* lactate dehydrogenase, *BUN* blood urea nitrogen, *Cr* creatinine, *CRP* C-reactive protein, *pCO*_*2*_ carbon dioxide partial pressure, *pO*_*2*_ oxygen partial pressure, *BE* base excess

**Table 2:** List of the bacteria that were isolated and results of antimicrobial susceptibility testing

Species	Bacteria count	Sampling site	Date of collection	Reporting date	Antimicrobial susceptibility testing		
					Susceptible	Intermediate	Resistant
*Clostridium perfringens*	2+	Closed abscess	POD2	POD6	ABPC, ABPC/SBT, CMZ, FMOX, IPM, MINO	LVFX	CLDM
	Small number	Drain	POD2	POD9	ABPC/SBT, IPM, MINO	ABPC, CMZ, FMOX, LVFX	CLDM
*Pseudomonas aeruginosa*	3+	Debris	POD14	POD17	PIPC, CPZ, CAZ, CZOP, CFS, CFPM, MEPM, AZT, S/C, TOB, AMK, LVFX, CPFX	IPM, GM	FOM
	3+	Open abscess	POD24	POD28			
*Pseudomonas aeruginosa* with metallo-β-lactamase	3+	Open abscess	POD33	POD38	PIPC, CAZ, CZOP, S/C, TOB, AMK	CPZ, CFS, CFPM, GM	IPM, MEPM, AZT, LVFX, CPFX, FOM
*Candida albicans*	2+	Debris	POD14	POD17	N/A	N/A	N/A
	2+	Open abscess	POD24	POD28	N/A	N/A	N/A
*Staphylococcus epidermidis*	N/A	CV catheter	POD8	POD15	VCM, TEIC, LZD	N/A	PCG, ABPC, MPIPC, CEZ, CTM, CPR, FMOX, IPM, GM, EM, CLDM, LVFX, ST, FOM
	N/A	Venous blood, CV catheter	POD14	POD17	VCM, TEIC	N/A	PCG, ABPC, MPIPC, CEZ, CTM, CPR, FMOX, IPM, GM, EM, CLDM, LVFX, ST, FOM
*Stenotrophomonas maltophilia*	3+	Open abscess	POD24	POD28	MINO, ST	LVFX	CAZ
	3+	Open abscess	POD33	POD38	MINO, ST	N/A	CAZ, LVFX
*Enterococcus casseliflavus*	3+	Open abscess	POD33	POD38	VCM, TEIC, LZD	N/A	LVFX, ABPC, PCG, EM

*POD* post-operative days, *ABPC* ampicillin, *ABPC/SBT* ampicillin/sulbactam, *CMZ* cefmetazole, *FMOX* flomoxef, *IPM* imipenem, *MINO* minocycline, *PIPC* piperacillin, *CPZ* cefoperazone, *CAZ* ceftazidime, *CZOP* cefozopran, *CFS* cefsulodin, *CFPM* cefepime, *MEPM* meropenem, *AZT* aztreonam, *S/C* sulbactam/cefoperazone, *TOB* tobramycin, *AMK* amikacin, *LVFX* levofloxacin, *VCM* vancomycin, *TEIC* teicoplanin, *LZD* linezolid, *ST* sulfamethoxazole–trimethoprim, *GM* gentamicin, *CLDM* clindamycin, *FOM* fosfomycin, *CPFX* ciprofloxacin, *PCG* benzylpenicillin, *MPIPC* oxacillin, *CEZ* cephazolin, *CTM* cefotiam, *CPR* cefpirome, *EM* erythromycin

## Discussion

Here, we report a fatal case of postoperative gas gangrene with very acute onset and rapid progression of the symptoms. This case report especially focuses on the risk factors, cause of infection, and treatment methods of postoperative gas gangrene.

Gas gangrene used to be frequent during war times, being related to weapon injuries [6]. In modern clinical practice, the various causes of gas gangrene have included “sterile” operations, intravenous infusion, intramuscular injection, and criminal abortion, etc. [7]. Even if a patient does not have any evident infectious causes, recent abdominal surgical intervention can also contribute to gas gangrene formation. Indeed, several cases of gas gangrene with *C. perfringens* after abdominal surgery have been recently reported [2, 3]. The onset of symptoms in these cases was 2 weeks and 2 days after the operation, respectively. To our knowledge, our case represents the most acute onset of postoperative gas gangrene with *C. perfringens* ever reported.

It is well known that both the existence of cancer cells and exposure to anesthetic agents can suppress the immune system, which in turn increases the risk of surgical site infection [8]. In addition, gas gangrene occurs more frequently in diabetics, alcoholics, immunosuppressed patients, IV drug users, and patients with peripheral vascular disease [9]. The risk of postoperative infection in our case may have been increased by the long operation and massive blood transfusion [8]. However, the patient had a past history of exposure to neither anticancer nor immunosuppressive agents. Moreover, she did not have any preexisting co-morbidities other than hyperthyroidism. Hence, she was not considered to have a particularly high risk for development of postoperative gas gangrene. Thus, the cause of fulminant infection with *C. perfringens* in this patient with no remarkable risk factors is unknown. Molecular typing of toxins and enzymes involved in the virulence of *C. perfringens* seems to be a powerful tool to clarify this issue. However, we did not assess the toxins and enzymes, which is a limitation of this case report.

We hypothesized that the source of infection in this case might have been the ileum used for bladder reconstruction. Urinary diversion via the bowel might contribute to contamination by bowel microbes [10]. In general, two conditions are necessary for the onset of gas gangrene: (1) the presence of clostridial spores, and (2) an area of tissue hypoperfusion caused by circulatory failure in a local area or by extensive soft tissue damage and necrotic muscle tissue. The occurrence of clostridial species in feces is not rare, a large number of clostridia having been found to be present in normal human feces (10^6^–10^9^/g feces) [7]. In particular, *C. perfringens* was reportedly detected in 33 % of healthy Japanese adults, and at a concentration of at least 10^3^/g feces [11]. These indicate that endogenous clostridial spores that probably existed in her ileum may have spread into the surgical wound. Abdominal enhanced CT images obtained 1 day after the operation (Fig. 2d, e) showed the presence of air-densities between the subcutaneous fat tissue and muscle layer in the vicinity of the drainage tubes. These CT images support our hypothesis that the drainage tube might have been the source of the *C. perfringens* infection.

Urgent surgical exploration and debridement of devitalized tissue are crucial for the treatment of gas gangrene. In addition, aggressive antibiotic treatment is also important. The first choice of antibiotics for Clostridium is penicillin [12]. However, we could not use penicillin because she had developed hypersensitivity responses to penicillin with shock at the age of approximately 20 years. Hence, we used IPM/CS and CLDM, which are considered the second choice for Clostridium. It was recently reported that CLDM resistant *C. perfringens* species are on the increase [1]. In this case, CLDM resistant *C. perfringens* was detected in the wound abscess. Therefore, we had to discontinue use of CLDM. Although *C. perfringens* was abolished by our intensive therapy, multidrug-resistant *Pseudomonas aeruginosa* caused multi-organ failure and, ultimately, death. Similar to what has been seen in many cases of severe sepsis, the secondary infection was likely due to a combination of neutropenia and an adverse reaction to broad spectrum antibiotics.

Despite remarkable progress of multidisciplinary therapeutic methods, including extensive surgical debridement, antibiotic coverage and HBOT, the morbidity and mortality rates of gas gangrene are still very high (up to 57 %) [13, 14]. Given this high mortality rate of gas gangrene, physicians should be aware that any patient could possibly develop gas gangrene after an operation.

## Abbreviations

ISP: isepamicin
IPM/CS: imipenem/cilastatin sodium
ICU: intensive care unit
APACHE II: acute physiology and chronic health evaluation II
SOFA: sequential organ failure assessment
CT: computed tomography
HBOT: hyperbaric oxygen therapy
PMX-DHP: polymyxin B-immobilized fiber column direct hemoperfusion
CHDF: continuous hemodiafiltration
CLDM: clindamycin
VCM: vancomycin
*C. perfringens*: *Clostridium perfringens*
